# Tribe Acalyptaini (*Hemiptera: Tingidae*: Tinginae) Revisited: Can Apomorphies in Secondary and Tertiary Structures of *18S rRNA* Length-Variable Regions (LVRs) Support Tribe Validity?

**DOI:** 10.3390/insects14070600

**Published:** 2023-07-03

**Authors:** Barbara Lis, Paweł J. Domagała, Jerzy A. Lis

**Affiliations:** Institute of Biology, University of Opole, 45-052 Opole, Oleska 22, Poland; canta@uni.opole.pl (B.L.); pdomagala@uni.opole.pl (P.J.D.)

**Keywords:** lace bugs, systematic position, phylogeny, nuclear rDNA, molecular apomorphies

## Abstract

**Simple Summary:**

The small subunit (SSU) of the nuclear ribosomal DNA that codes *18S rRNA* is one of the most frequently sequenced genes in phylogenetic analyses of true bugs (Hemiptera: Heteroptera). However, no studies have been identified that use this method with lace bugs (Tingidae). Furthermore, the secondary and tertiary structures of the *18S rRNA* have not been described nor used to interpret relationships among lace bug taxa. The number of nucleotides and shapes of the *18S rRNA* length-variable regions (LVRs) have been confirmed to be phylogenetically informative; therefore, we verified their usefulness in resolving the validity issues within the Acalyptaini tribe.

**Abstract:**

The lace bug tribe Acalyptaini (Tingidae: Tinginae) includes five genera, *Acalypta*, *Derephysia*, *Dictyonota*, *Kalama,* and *Recaredus,* and it was recently resurrected based on morphological and karyological characters. We aimed to validate the distinctiveness of this tribe using *18S rDNA* sequences, which have not been used in previous Tingidae phylogenomic studies. Our results confirmed the monophyly of the tribe. Moreover, the monophyly of the subfamily Cantacaderinae and its basal position within the family Tingidae were indicated, as well as the position of the tribe Litadeini as sister to all other Tinginae. In addition, we attempted to determine the apomorphic morpho-molecular characters in the secondary and tertiary structures of length-variable regions of the *18S rRNA* sequences of the analysed species. The results showed that two LVRs (LVR X and LVR L) of the hypervariable region V4 exhibited significant variability in the number of nucleotides and could be considered for apomorphic recognition.

## 1. Introduction

The family Tingidae (commonly called lace bugs due to their lacelike body appearance) is currently divided into three subfamilies: Vianaidinae, Cantacaderinae, and Tinginae [1]. The Vianaidinae subfamily is a small group, which has not been divided into tribes [1,2], and the Cantacaderinae contain several relatively uncontested tribes [1,3,4]. The internal classification of contemporary Tinginae has not been fully established [1,5,6,7,8]; however, four tribes are usually recognised, Tingini Laporte, 1832, Litadeini Drake and Ruhoff, 1965, Phatnomini Drake and Davis, 1960, and Ypsotingini, Drake and Ruhoff, 1965.

Recently [9], the phylogenetic affinity of the morphological and karyological characters of the genera *Acalypta* Westwood, 1840, *Dictyonota* Curtis, 1827, *Kalama* Puton, 1876, and *Derephysia* Spinola, 1837 were demonstrated through a comparative analysis, and these genera were then united into a single tribe under the resurrected valid name Acalyptini (Acalyptaini; ICZN Case 3813) [10]. Previously, these genera had been classified into three tribes: Acalyptini Blatchley, 1926, or Tingini Laporte, 1832, for *Acalypta* and Ypsotingini, Drake and Ruhoff, 1965, for *Dictyonota*, *Derephysia,* and *Kalama* [1,5,7,11].

Moreover, comparative cytogenetic studies of lace bugs have confirmed that the same chromosome numbers and sex chromosome systems exist in these genera, and these differ from those of the species representing the tribe Tingini [12]. Morphological character and genital structure analyses have recently resulted in the genus *Recaredus* Distant, 1909, which was allocated to the tribe Ypsotingini, to be included in the tribe Acalyptaini [13]. Thus, the tribe Acalyptaini presently contains five genera: *Acalypta*, *Derephysia*, *Dictyonota*, *Kalama,* and *Recaredus* [11].

Species of these genera are mainly Holarctic in distribution [1,5,7,9], but some have been identified in the tropical regions of Africa and Asia [5,7,11,13].

The external morphological characters that best identify the tribe [9,11,13] are: (1) a head with two (frontal) or four (frontal and occipital) spines or tubercles (the median spine or tubercle is lacking); (2) buccal laminae, which are not closed anteriorly; (3) a pronotum with slightly oblique paranota, not reflexed and not cyst-forming; (4) a flat posterior process of the pronotum; and (5) a metathoracic scent gland opening, which is absent of a peritreme. Moreover, the structure of the aedeagus in all genera of this tribe (*Acalypta*, *Derephysia*, *Dictyonota*, *Kalama,* and *Recaredus*) differs distinctly from that found in other genera of Tinginae tribes and other Tingidae subfamilies [11,13,14]. Two aedeagal structures, the bifurcate *ductus seminis* and small *endosomal diverticula*, are considered the most important genital characters for identifying species of this tribe [11,13,14]. 

Additional essential features suggesting close phylogenetic affinities for the four genera classified within the tribe Acalyptaini (*Acalypta*, *Derephysia*, *Dictyonota* and *Kalama*) concern the sex chromosome system [9,12]. According to Golub and Golub [9] and Golub et al. [12], the tribe-specific karyotype is 2 n = 12 + X0/XX (male/female), which differs significantly from the karyotype described for species of the tribe Tingini, which is 2 n = 12 + XY/XX (male/female) (these data are summarised in [9] and [12]).

Identification keys have been prepared for these four genera [9] and all five genera within this tribe (*Acalypta*, *Derephysia*, *Dictyonota*, *Kalama*, and *Recaredus*) [11]. Photographs of the total habitus of species representing the Acalyptaini genera have been presented by Golub and Golub [9] and Lis et al. [11].

The only previous molecular data analysis of relationships within the entire Tingidae identified [8] utilised sequences of four mitochondrial DNA genes (*COI*, *Leu-tRNA*, *COII* and *16S*), with only one of these representing nuclear DNA (*28S rDNA*). Therefore, we attempted to verify whether an additional nuclear DNA gene (*18S rDNA*) sequence analysis would confirm the close relationship of the genera currently included in the tribe Acalyptaini.

In addition, based on recent studies on the secondary and tertiary structures of RNA encoded by this gene [15] in Heteroptera, we tested whether morpho-molecular apomorphies in these structures could be included in the analysis of relationships among taxa within the family Tingidae.

## 2. Materials and Methods

### 2.1. Selection of Taxa

In this study, 22 terminal taxa were included in the analysis, with 19 forming the ingroup and three as the outgroup (Table S1). The ingroup contained species of the family Tingidae, including three with *18S rDNA* complete sequences that were obtained from GenBank (Table S1), and 16 which were newly sequenced in this study (Table S2). Three species of other cimicomorphan families were selected as the outgroup (Table S1). Two of these represented the family Miridae, the sister group of Tingidae within the superfamily Miroidea [1,4,16], and one belonged to the superfamily Naboidea, which is usually considered a sister to Miroidea [4,16].

Taxa names, geographic origins, collector names, University of Opole (Poland) sample numbers (if applicable), and accession numbers for sequences we deposited into GenBank, and those obtained directly from GenBank are provided in Tables S1 and S2.

### 2.2. DNA Extraction

Ethanol-preserved specimens were used for genomic DNA extraction, except for those of *Recaredus rex* Distant, 1909, which were obtained from dry museum specimens. The total genomic DNA was extracted from the thorax muscle tissues of each species using the DNeasy Tissue Kit (QIAGEN Inc., Santa Clara, CA, USA), following the manufacturer’s protocol. The remains of the specimens were then inserted into tubes with 96% ethanol and placed in a deep freezer at the Institute of Biology, University of Opole (for the University of Opole sample numbers, see Table S2).

### 2.3. PCR Amplification, Purification and Sequencing

The PCR amplification for *18S rDNA* was performed using a 25 μL reaction volume consisting of 1 μL of DNA template, 1× reaction buffer, 0.5 μL of each primer, 200 μm dNTPs mix, and 0.02 U/μL of HiFiTaq^®^ DNA Polymerase [17]. The length of the *18S rDNA* exceeded that required for a single amplification; therefore, we used two sets of primers. The first included three primer pairs: 1F-5R, 3F-18Sbi, and 5F-9R [18,19], and the second consisted of two primer pairs: Ns1-18SP3 and 18SP5-Ns8 [20,21,22,23] (Table S3).

The PCR reactions were conducted using an Eppendorf Master Thermocycler, following the procedure described by Lis et al. [17], with 36 cycles of denaturation at 93 °C for 1 min, annealing at 59 °C for 1 min and extension at 72 °C for 40 s, with an initial denaturation step of 93 °C for 2 min and a final extension step of 72 °C for 5 min. The quality of the final PCR products was evaluated by 1% agarose gel electrophoresis. The successful samples were purified using the Qiaquick PCR Purification Kit (QIAGEN Inc.) and eluted in 30 μL of elution buffer.

All experimental PCR runs were completed concurrent with those of the negative controls (without templating DNA). Purified amplicons were sequenced in the Health Care Center GENOMED (Warsaw, Poland) with appropriate sequencing primers. The obtained sequences were verified using BLAST searches to certify that the results were not those of contaminants. All newly obtained DNA sequences were deposited in GenBank (OR022068–OR022083), and their accession numbers are provided in Tables S1 and S2.

### 2.4. Phylogenetic Analysis

Sequences were aligned using ClustalW (with default parameters) in the MEGAX software [24] and then truncated at both ends to avoid the influence of missing data from incomplete sequences.

A Maximum Likelihood tree was generated using IQ-TREE [25] on the web server [26] with 10,000 replications of the Ultrafast Bootstrap method [27]. The obtained tree was visualised and edited using the online tool iTOL v5 [28] and prepared for publication with CorelDRAW 21.

### 2.5. Reconstruction of Secondary Structures

The secondary structure of the *18S rRNA* for each species was constructed according to the models provided for Heteroptera [15,22,29]. The three hypervariable regions (V2, V4, V7), which are considered crucial for recovering the phylogenetic relationships among higher-level Heteroptera taxa [15,22,29], were analysed.

**Figure 1:**
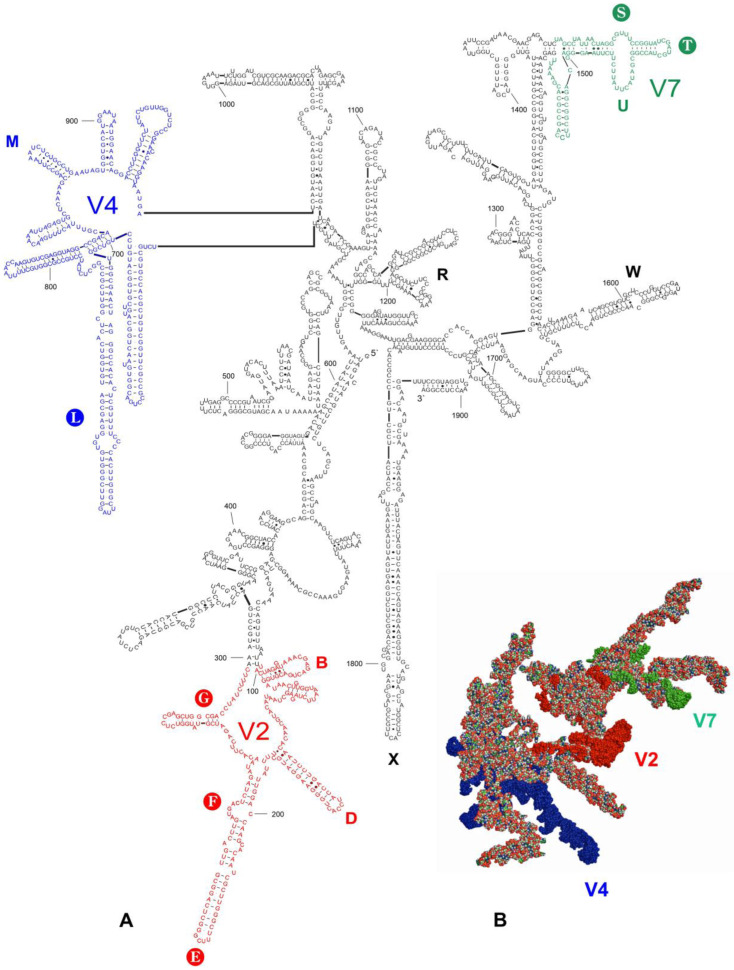
*18S rRNA* of *Acalypta sauteri*. (**A**) Secondary structure model. The bases marked in colour represent the hypervariable regions (V2—red, V4—dark blue, V7—green). Thirteen length–variable regions (LVRs) are labelled as capital letters B to W in colours analogous to the base colours representing the hypervariable regions or other sequences’ regions. The capital letters in filled circles indicate six LVRs (E, F, G, L, S, and T) that can serve as molecular synapomorphies or autapomorphies in analyses. Base pairing is shown as follows: standard canonical pairs are lines (G–C, A–U), wobble G:U pairs are dots (G·U), A:G or A:C pairs are open circles (A; G, A; C), and other non-canonical pairs are filled circles (e.g., U and U, A and A). (**B**) Tertiary structure model. The fragments marked in colour represent the hypervariable regions (V2—red, V4—dark blue, V7—green).

Thirteen length-variable regions (LVRs) were identified in the heteropteran *18S rRNA* secondary structure models [15,22,29], and those which could potentially serve as morpho-molecular apomorphies (synapomorphies or autapomorphies) were considered for further analysis: three LVRs (E, F, G) in the V2 region, one (L) in the V4 region, and two (S, T) in the V7 region [15,22,29] (Figure 1).

LVR L was the longest and most variable region, and it presented the most appropriate length-variable region for phylogenetic relationship analyses [15,22,29]. Therefore, it was thoroughly examined, and its secondary structures were predicted using the computer program RNAstructure ver. 6.3 [30]. The three-step procedure described by Lis [15] was applied to the comparative sequence analysis. RNAstructure ver. 6.3 suggested a species that exhibited a secondary structure common to two or more sequences, and this was considered the “consensus species” for these sequences [15].

The hypervariable region numbering, the numbering system for the length–variable regions (LVRs), and the nucleotide numbering of the entire gene sequences followed that of Yu et al. [23], Wu et al. [29], and Lis [15]. Subdividing of the secondary structures of LVR L into subregions was performed according to Lis [15].

All secondary structures were visualised using RNAstructure ver. 6.3 [30].

### 2.6. Prediction of Tertiary Structures

The *18S rRNA* gene tertiary structures were predicted with 3dRNA v2.0 Web Server (http://biophy.hust.edu.cn/new/3dRNA, accessed on 8 May 2023) [31]. An optimisation procedure and the ‘ProbKnot’ method were selected for prediction, similar to those used for the *18S rRNA* analyses of shield bugs (Pentatomoidea) [15].

LVR L tertiary structures were predicted using RNAComposer (http://rnacomposer.ibch.poznan.pl, accessed on 10 May 2023), which is a fully automated RNA structure modelling server [32,33]. Twenty 3D RNA models were generated for each LVR sequence, and the best model with the lowest free energy was selected for analysis. The tertiary structural images were visualised using PyMol software ver. 2.4.0 [34].

### 2.7. Morpho-Molecular Structures Potentially Serving as Apomorphies

The concept of morpho-molecular apomorphies (autapomorphies and synapomorphies) of nucleotide sequences in the predicted *18S rRNA* secondary structures followed that of Lis [15], Yu et al. [23], Xie et al. [35], and Ouvrard et al. [36].

To recognise the apomorphies in the predicted LVR tertiary structures of the analysed *18S rRNA* sequences, a two-step procedure recently proposed by Lis [15] was applied. Only tertiary structures which had their distinctness confirmed at the level of secondary structures were considered apomorphic [15].

## 3. Results

### 3.1. Sequence Analysis and Tree Topology

The *18S rDNA* genes of 16 of the 17 species analysed were successfully amplified and sequenced; only the extraction and amplification of the dry museum specimen of *Recaredus rex* Distant, 1909 failed (Table S2).

The final *18S rDNA* alignment contained 1931 sites. The number of conserved and variable sites were 1536 and 383, respectively, while 211 sites were parsimony-informative, and 172 were singletons. The alignment file used for the phylogenetic analysis is provided in the Supplementary Materials (File S1).

**Figure 2:**
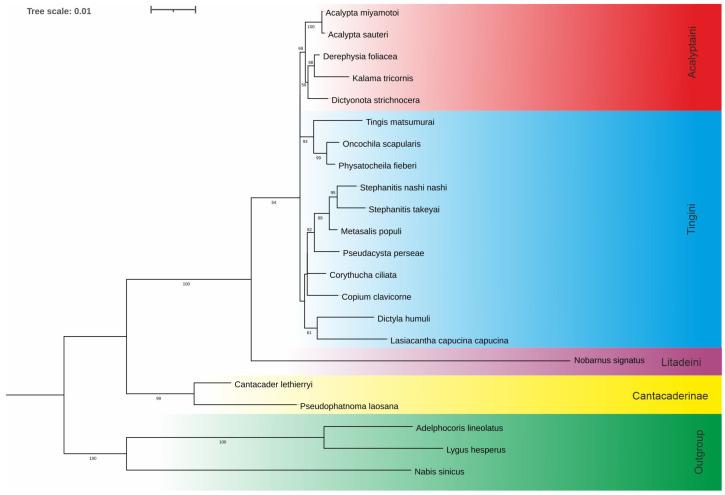
Phylogenetic tree based on a Maximum Likelihood analysis of the *18S rDNA*. Node labels—Ultrafast Bootstrap values (see Section 2), and the Bootstrap values over 50% are shown next to the branches, scale bar—number of substitutions per site.

ModelFinder in the IQ-TREE [24] has tested 88 DNA models for this set of sequences, and the TNe + I + G4 substitution model was chosen as the best fit according to the Bayesian Information Criterion. The IQ-TREE generated 98 initial parsimony trees; the ML consensus tree is shown in Figure 2.

Our analyses showed that the tribe Acalyptaini conceived by Golub et al. [9] was a monophyletic group with a node support of 68% ML bootstrap value (MBLv) (Figure 2). However, the species representing the tribe Tingini formed two independent lineages on the tree, the first including species of the *Tingis*, *Oncochila,* and *Physatocheila* genera, and the second consisted of *Stephanitis*, *Metasalis*, *Pseudacysta*, *Corythucha*, *Copium*, *Dictyla,* and *Lasiacantha* species.

*Nobarnus signatus* (Litadeini) was indicated as belonging to the sister group to Acalyptaini + Tingini, and two species of Cantacaderinae were recovered as a strongly supported monophyletic clade with a node support of 99% MBLv. What is essential is that the clade was retrieved as a sister to all species of the subfamily Tinginae. The latter clade (including Acalyptaini, Tingini, and Litadeini) had a ML bootstrap value of 100% (Figure 2).

### 3.2. Secondary Structure Models

The secondary structure models of the entire *18S rRNA* gene were predicted for 15 species, including all consensus species (File S2). The prediction included four species of the tribe Acalyptaini, seven of Tingini, one of Litadeini, one of the subfamily Cantacaderinae, and three species of the outgroup. Although the secondary structure models were similar in their general outlines, local differences were found within certain hypervariable regions (V) and length–variable regions (LVRs) (File S2). The secondary structure model of *18S rRNA* of *Acalypta sauteri* Drake, 1942, showing the positions of these regions, is presented in Figure 1.

The nucleotides in the *A. sauteri* sequence formed 584 pairs (61.2% of all nucleotides in the secondary structure model), with the standard canonical pairs (G–C and A–U) as the most common (450 pairs, 77.1%). The wobble G:U pairs were approximately six times less commonly formed than the standards between the paired nucleotides (83 pairs, 14.2%). The A:G, A:C and other non-canonical pairs were observed rarely (51 pairs, 8.7%). The number of nucleotides that formed particular pair types varied insignificantly among the studied sequences (i.e., ±1.0–1.5% in the standard canonical, ±1.5–3.0% in the wobble G:U, and ±4.5–7.5% in all other non-canonical pairs). This range of variability was consistent with those of previous data for other *Heteroptera* species [23,29,37].

The alignment results of the entire *18S rRNA* sequences analysed (File S1) confirmed the existence of the three hypervariable regions (V2, V4, and V7), which have been described in Heteroptera [15,23,29,37]. The sequence length of the V4 region was highly diverse (299–323 nucleotides), whereas those of the V2 and V7 regions were less variable (198–202 and 90–91 nucleotides, respectively) (File S1, Table 1). However, when considering all analysed species (not only the consensus species), the V4 hypervariable region sequence length (Files S1, Table 1) was stable within the Acalyptaini (at 321 nucleotides) and the Cantacaderinae (at 299 nucleotides). In contrast, this region was highly variable within the Tingini (305–321 nucleotides).

**Figure 3:**
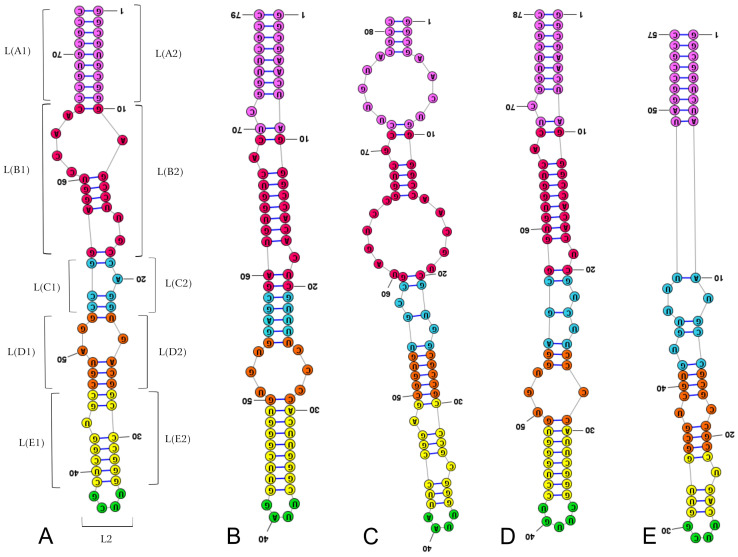
Secondary structure models of the length–variable region L. (**A**) *Adelphocoris lineolatus* (outgroup). (**B**) *Acalypta sauteri* (Tinginae: Acalyptaini). (**C**) *Nobarnus signatus* (Tinginae: Litadeini). (**D**) *Tingis matsumurai* (Tinginae: Tingini). (**E**) *Cantacader lethierryi* (Cantacaderinae). Specific subregion bases are marked in the same colour.

The positions of the LVRs within the gene sequences are shown for *Acalypta sauteri* (Figure 1) and all analysed consensus species (in File S2). The LVR G, which has been detected in some heteropterans [15,29], was absent in all analysed species. The five LVRs (M, T, U, R, and W) displayed the same number of nucleotides for each region, while six others (B, D, E, F, S, and X) exhibited only insignificant variations in length (one to three nucleotide differences) (Table 2). All 11 of these LVRs were short (three to thirteen nucleotides). In contrast, LVR L was relatively long (from 57 to 81 nucleotides) and showed distinct variations in the sequence lengths (Table 2). Therefore, as suggested in the recent analyses of the *18S rRNA* secondary structures in the heteropteran superfamily Pentatomoidea [15], this region was subdivided into subregions to compare the homologous fragments in analysed sequences (Figure 3, Figure S1). The number of nucleotides for each subregion resulting from this comparative analysis is provided in Table 3.

Two subregions, L2 and LA (LA1 + LA2), showed little variability (a single nucleotide). All remaining subregions were variable, with nucleotide numbers ranging from 19–24 in LB, 7–12 in LC, 10–14 in LD, and 9–16 in LE (Table 3).

### 3.3. Tertiary Structure Models

**Figure 4:**
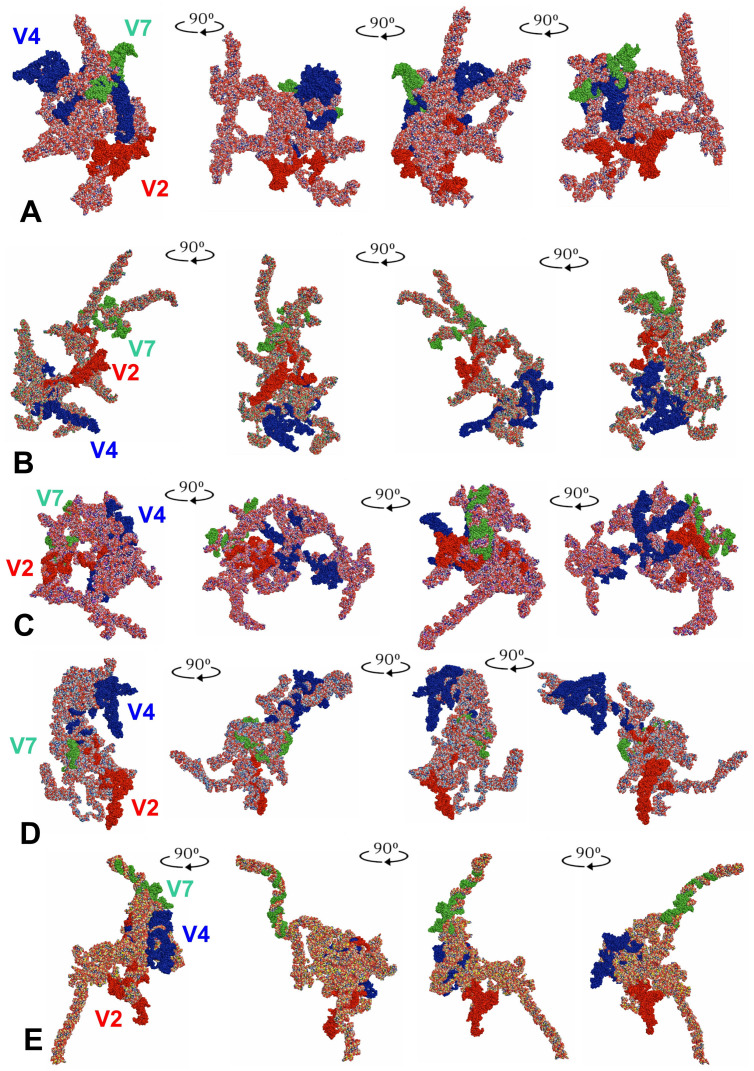
Tertiary structure models of the *18S rRNA*. (**A**) *Adelphocoris lineolatus* (outgroup). (**B**) *Acalypta sauteri* (Tinginae: Acalyptaini). (**C**) *Nobarnus signatus* (Tinginae: Litadeini). (**D**) *Tingis matsumurai* (Tinginae: Tingini). (**E**) *Cantacader lethierryi* (Cantacaderinae). The hypervariable regions are marked in red (V2), dark blue (V4), and green (V7).

The tertiary structure models of the entire *18S rRNA* genes were predicted for all five consensus species (Figure 4).

**Figure 5:**
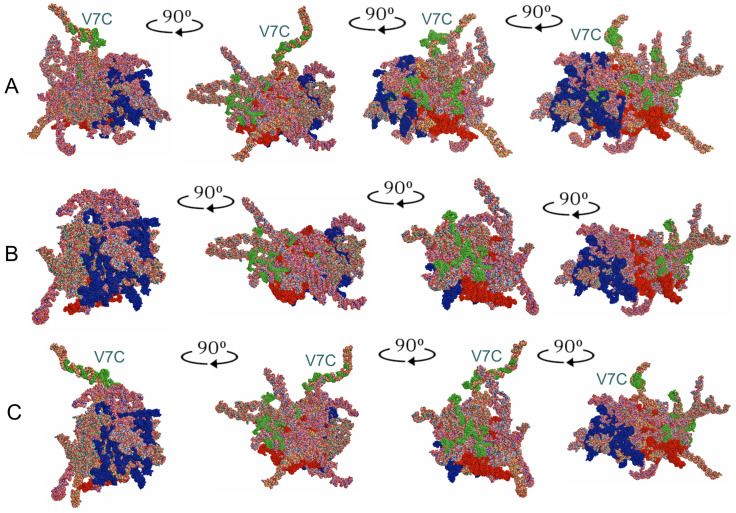
*18S rRNA* tertiary structure models. (**A**) Combined for all five consensus species and aligned to the outgroup (*Adelphocoris lineolatus*) sequence. (**B**) Combined for three consensus species of the subfamily Tinginae and aligned to *Acalypta sauteri* sequence. (**C**) Combined for four consensus species of Tingidae (Tinginae and Cantacaderinae) and aligned to *Cantacader lethierryi* sequence. The hypervariable regions are marked in red (V2), dark blue (V4), and green (V7). V7C—hypervariable region V7 in *Cantacader lethierryi*.

When the tertiary structures of the consensus species were aligned (Figure 5A), the three species, representing the subfamily Tinginae (*A. sauteri, N. signatus,* and *T. matsumurai*) appeared comparable (Figure 5B). This similarity involved the location of the hypervariable regions V2, V4, and V7. However, the general shape of the *18S rRNA* tertiary structure for *Cantacader lethierryi* of the subfamily Cantacaderinae differed significantly, especially when the V7 hypervariable region was considered (Figure 5C).

**Figure 6:**
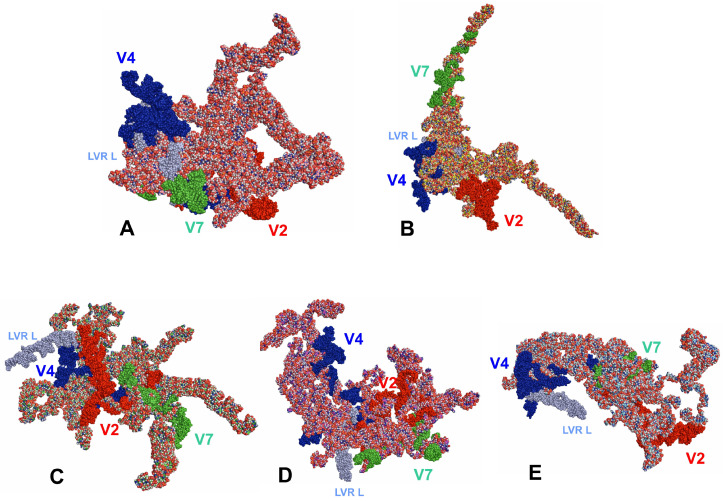
The LVR L position (marked in light blue) within the hypervariable region V4 in the tertiary structure models of the *18S rRNA* gene. (**A**) *Adelphocoris lineolatus* (outgroup). (**B**) *Cantacader lethierryi* (Cantacaderinae). (**C**) *Acalypta sauteri* (Tinginae: Acalyptaini). (**D**) *Nobarnus signatus* (Tinginae: Lita-deini). (**E**) *Tingis matsumurai* (Tinginae: Tingini). The hypervariable regions are marked in red (V2), dark blue (V4), and green (V7). All sequences are aligned to the outgroup (*A. lineolatus*) sequence.

LVR L, which was the longest segment (57 to 81 nucleotides) of the hypervariable region V4, differed in its level of visibility. This region was indistinct and mainly hidden in the core of the entire tertiary structure in *Adelphocoris lineolatus* (the outgroup) and *Cantacader lethierryi* (the subfamily Cantacaderinae of Tingidae) (Figure 6A–B). However, in three consensus species of the subfamily Tinginae (*Acalypta sauteri*, *Nobarnus signatus* and *Tingis matsumurai*), the LVR L was well recognisable (Figure 6C–E).

**Figure 7:**
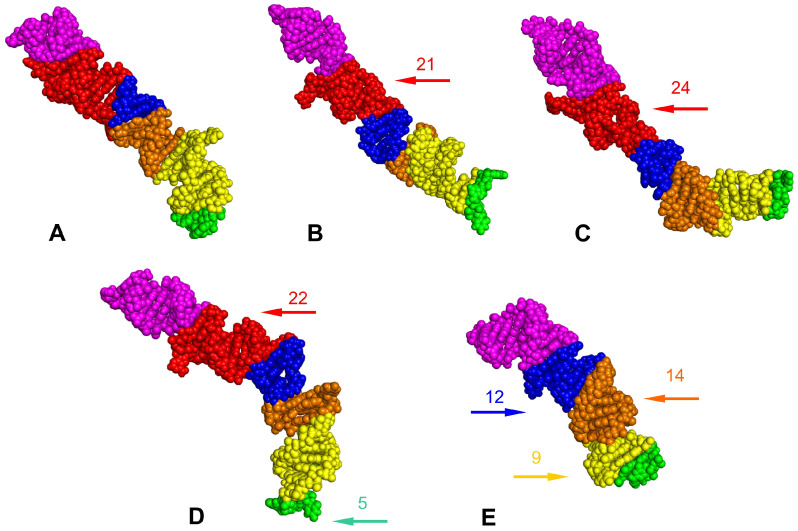
The predicted tertiary structure models of the LVR L. (**A**) *Adelphocoris lineolatus* (outgroup). (**B**) *Acalypta sauteri* (Tinginae: Acalyptaini). (**C**) *Nobarnus signatus* (Tinginae: Litadeini). (**D**) *Tingis matsumurai* (Tinginae: Tingini). (**E**) *Cantacader lethierryi* (Cantacaderinae). The LVR subregions are marked in the appropriate colour: L (A1 + A2) in magenta, L (B1 + B2) in red, L (C1 + C2) in blue, L (D1 + D2) in orange, L (E1 + E2) in yellow, and L2 in green. The arrows, corresponding in colour to the particular subregion, indicate the fragments that can serve as potential morpho-molecular derived characters (autapomorphies). All sequences are aligned to the outgroup (*A. lineolatus*) sequence. The numbers above the arrows show the autapomorphic number of nucleotides in the subregion.

**Figure 8:**
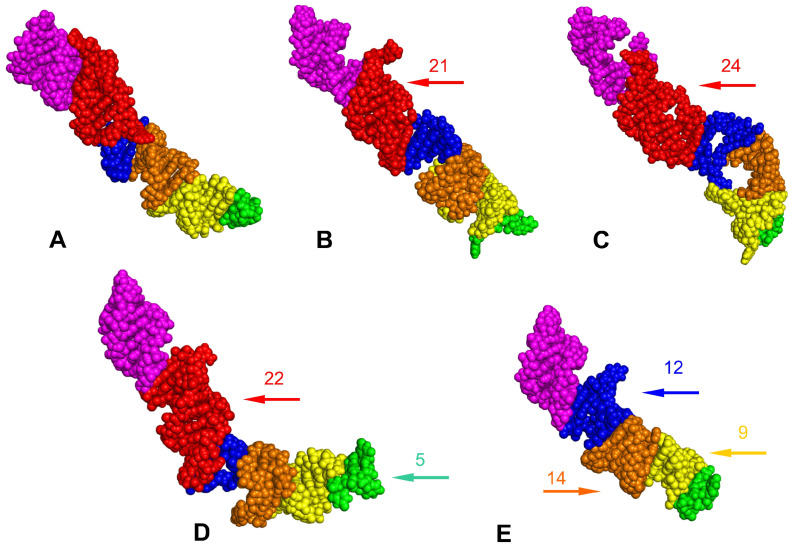
The predicted tertiary structure models of the LVR L. (**A**) *Adelphocoris lineolatus* (outgroup). (**B**) *Acalypta sauteri* (Tinginae: Acalyptaini). (**C**) *Nobarnus signatus* (Tinginae: Litadeini). (**D**) *Tingis matsumurai* (Tinginae: Tingini). (**E**) *Cantacader lethierryi* (Cantacaderinae). The LVR subregions are marked in the appropriate colour: L (A1 + A2) in magenta, L (B1 + B2) in red, L (C1 + C2) in blue, L (D1 + D2) in orange, L (E1 + E2) in yellow, and L2 in green. The arrows corresponding in colour to the particular subregion indicate the fragments that can serve as potential morpho-molecular derived characters (autapomorphies). All sequences are aligned to *A. sauteri* sequence. The numbers above the arrows show the autapomorphic number of nucleotides in the subregion.

The predicted tertiary structures of the LVR Ls for all five consensus species are presented in Figure 7 and Figure 8, with all subregions recovered in four of these (*A. lineolatus*, *A. sauteri*, *N. signatus* and *T. matsumurai*). For *C. lethierryi*, subregion LB was missing what can be considered its autapomorphy. The other fragments that could serve as morpho-molecular autapomorphies are indicated by the arrows that are colour-coded to the particular LVR L subregion.

## 4. Discussion

### 4.1. 18S rRNA Secondary and Tertiary Structures of Tingidae

The *18S rRNA* secondary structure models predicted for the four Tingidae consensus species were similar (File S2). Differences were identified within certain hypervariable regions (V2, V4 and V7) (Table 1), especially when modifications in the LVRs were considered (Table 2, Figure 3). Among the LVRs analysed, LVR L appeared the most variable, which corroborates the results of previous studies on *18S rRNA* secondary structures in Heteroptera [15,23,29].

Despite similarities among secondary structures of the analysed species, their predicted tertiary structures differed, sometimes substantially (Figure 4 and Figure 5). In particular, the specific tertiary structure of *Cantacader lethierryi* of the subfamily Cantacaderinae differed significantly from those of all other species representing the subfamily Tinginae (Figure 4, Figure 5 and Figure 6).

Our results support the recent opinion [15] that probable in vivo tertiary configurations of the *18S rRNA* are not predictable using existing software (3dRNA v2.0 Web Server). However, small nucleolar RNAs (snoRNAs) activities could have modified the ribosomal RNA tertiary structures [38,39,40,41,42]. The impact of these activities on such structures has not been identified in heteropteran studies; therefore, further research is required in this area [15].

### 4.2. Potential Apomorphies in Secondary and Tertiary Structures of LVRs

Our analysis demonstrated that the unique nucleotide numbers of two LVRs potentiate these regions as autapomorphies for a particular taxon.

LVR X had a unique number of nucleotides (three to four), which is a potential autapomorphy for the tribe Acalyptaini (Table 2, Figure 1). All other analysed species have five to six nucleotides for this LVR (Table 2, File S2).

However, the most effective method for recovering apomorphic morpho-molecular characters was the comparative analyses of LVR L secondary and tertiary structures (Figure 3, Figure 6, Figure 7 and Figure 8). These results showed the occurrence of several synapomorphies and autapomorphies related to the number of nucleotides in specific subregions (Table 3).

The nineteen nucleotides in the LA subregion, eight in the LE subregion, and ten in the LD subregion can be considered synapomorphies for the Acalyptaini, Litadeini and Tingini of the subfamily Tinginae (Table 3). In addition, an equal number of nucleotides (16) in the LE subregion for Acalyptaini and Litadeini could indicate the synapomorphy of these tribes.

Among the analysed taxa, Cantacaderinae had the highest number of autapomorphies (four) in the specific subregions (except for L2 and LA). Tingini had two autapomorphies (in L2 and LB), while a single autapomorphy was revealed for Acalyptaini and Litadeini (Table 3). The most noteworthy autapomorphy was the absence of the entire subregion LB in the Cantacaderinae; such an extensive deletion in the *18S rDNA* sequence has not been recorded in Heteroptera. Strict morpho-molecular autapomorphies in the LVR L tertiary structures were found in four subregions, LB, LC, LD, and L2 (Figure 7 and Figure 8). However, most morpho-molecular autapomorphies involved the LB subregion (Figure 7 and Figure 8) and unique nucleotide numbers for certain tribes (21 for Acalyptaini, 22 for Tingini and 24 for Litadeini). A single morpho-molecular autapomorphy was recovered for the four other subregions (Figure 7 and Figure 8): one in L2 for the Tingini, and one each in LC, LD, and E for Cantacaderinae (Figure 7 and Figure 8). These results indicate that the subfamily Cantacaderinae exhibited the highest number of morpho-molecular autapomorphies in the secondary and tertiary structures of the *18S rRNA*. In addition, these results agree with our phylogenomic analysis, which identified the Cantacaderinae as sister to all other Tingidae used by us (Figure 2). Unfortunately, we could not study any representative of the subfamily Vianaidinae, usually considered the sister group to Tingidae (sensu stricto) (Tinginae + Cantacaderinae), based on morphological analyses [2,43,44].

Because the species of Vianaidinae were never included in any known molecular phylogenetic studies, we must bear in mind that the sister relationship between Cantacaderinae and Tinginae presented here cannot be fully supported without the addition of the Vianaidinae into the molecular phylogenetic analysis of the entire Miroidea.

### 4.3. Identity and Systematic Position of the Tribe Acaltyptaini within the Subfamily Tinginae

Although the tribe Acalyptaini has only recently been validated [9], three of its genera (*Acalypta*, *Dictyonota,* and *Kalama*) have already been included in the first and only phylogenomic analysis of the entire family Tingidae [8]. Five loci were used, including four from mitochondrial genes (*COI*, *Leu-tRNA*, *COII,* and *16S rRNA*) and one from a nuclear gene (*28S rRNA*). That study [8] indicated, among others, that these three genera (*Acalypta*, *Dictyonota,* and *Kalama*) form a monophyletic clade. However, the clade was identified a part of the broadly conceived Tingini [8], which is contrary to our present findings.

Our results confirmed the monophyly of the tribe Acalyptaini, based on an analysis of *18S rDNA* sequences, which has not been used in previous phylogenomic studies of Tingidae [8]. We could not include the DNA sequences of *Recaredus rex*, which was recently included in the tribe Acalyptaini [11], due to the lack of freshly collected specimens. Therefore, placing this genus within the tribe must be based only on morphological characters [11].

In addition, the present study indicated that the tribe Tingini was not monophyletic. The Litadeini, placed within the tribe Tingini by Guilbert et al. [8], was suggested in our study as sister to the clade, which consisted of the Acalyptaini and Tingini. In both molecular analyses [8], in the present study, the tribe Litadeini was represented only by New Caledonian endemic taxa (two genera with three species, and one genus with a single species, respectively). Moreover, the tribe range is considered pantropical [7]. Therefore, all these could be a reason for explaining the different Litadeini position on the phylogenetic tree of Tingidae obtained in both surveys [8] in the present study.

## 5. Conclusions

1. The results of the present molecular analyses (phylogenetic and structural) validated the recognition of the tribe Acalyptaini within the subfamily Tinginae.
2. The monophyly of the subfamily Cantacaderinae and its basal position within the family Tingidae were indicated, as well as the position of the tribe Litadeini as sister to all other Tinginae.
3. The structural analysis of the predicted tertiary structures of the entire *18S rRNA* confirmed the proposed hypothesis that the in vivo configuration of this gene is likely not predictable using only secondary structure models and existing software. However, this may result from small nucleolar RNAs (snoRNAs) activities that can affect changes in the tertiary structures of ribosomal genes.
4. The results showed that two LVRs (LVR X and LVR L) of the hypervariable region V4 exhibited significant variability in the number of nucleotides and could be considered for apomorphic recognition.
5. LVR L appeared to be the most appropriate for phylogenetic relationship analysis within the family Tingidae when considering the secondary and tertiary structure models suitable for identifying morpho-molecular apomorphies.
6. The subfamily Cantacaderinae exhibited the highest number of morpho-molecular autapomorphies in the secondary and tertiary structures of the *18S rRNA*. In particular, the absence of the entire subregion LB in this subfamily is the first example of such an extensive deletion in the *18S rDNA* sequence in Heteroptera.
7. The tertiary structure of the *18S rRNA* exhibited evolutionary properties, which were not detectable in the primary or secondary structures. Therefore, the results of rRNA tertiary structure analyses for phylogenetic considerations are promising. Therefore, including the methods of rRNA tertiary structure analyses in the phylogenetic evaluations in other groups of Heteroptera is strongly suggested.

## Figures and Tables

**Table 1:** The number of nucleotides of the hypervariable regions V2, V4, and V7 in the *18S rRNA* of the analysed taxa. For the ‘consensus species’ definition, see Section 2.

Taxon Group	Consensus Species	Number of Nucleotides		
		V2	V4	V7
Outgroup	*Adelphocoris lineolatus* (Goeze, 1778)	198	317	91
Tinginae: Acalyptaini	*Acalypta sauteri* (Drake, 1942)	200	321	90
Tinginae: Litadeini	*Nobarnus signatus* (Distant, 1920)	202	323	90
Tinginae: Tingini	*Tingis matsumurai* (Takeya, 1962)	200	320	90
Cantacaderinae	*Cantacader lethierryi* (Scott, 1874)	199	299	90

**Table 2:** The nucleotide numbers of the LVRs in the *18S rRNA* of the analysed taxa. The autapomorphy for Acalyptaini is indicated in red.

Taxon Group	Number of Nucleotides												
	B	D	E	F	G	L	M	S	T	U	R	W	X
outgroup	12	6	5	4	0	60–78	4	6	7	13	5	3	6
Tinginae: Acalyptaini	11	5	4	5	0	79	4	5	7	13	5	3	3–4
Tinginae: Litadeini	11	4	5	5	0	81	4	5	7	13	5	3	6
Tinginae: Tingini	11	5	4–5	5	0	63–79	4	5	7	13	5	3	5–6
Cantacaderinae	11	5	5	4	0	57	4	5	7	13	5	3	5

**Table 3:** The nucleotide numbers of the subregions of the LVR L. The autapomorphies are indicated in red, and the synapomorphies are indicated in green.

Taxon Group	Consensus Species	Total Length	Number of Nucleotides of the LVR L Subregions					
			L2	LA (A1 + A2)	LB (B1 + B2)	LC (C1 + C2)	LD (D1 + D2)	LE (E1 + E2)
outgroup	*Adelphocoris lineolatus*	74	4	18 (9 + 9)	19 (10 + 9)	7 (3 + 4)	11 (6 + 5)	15 (8 +7)
Acalyptaini	*Acalypta sauteri*	79	4	19 (10 + 9)	21 (11+ 10)	8 (4 + 4)	10 (5 + 5)	16 (8 + 8)
Litadeini	*Nobarnus signatus*	81	4	19 (10 + 9)	24 (13 + 11)	8 (4 + 4)	10 (5 + 5)	16 (8 + 8)
Tingini	*Tingis matsumurai*	78	5	19 (10 + 9)	22 (11 + 11)	8 (3 + 5)	10 (6 + 4)	14 (7 + 7)
Cantacaderinae	*Cantacader lethierryi*	57	4	18 (9 + 9)	0	12 (7 + 5)	14 (7 + 7)	9 (4 + 5)

## Data Availability

The data presented in this study are available in the article and supplementary material.

## Supplementary Materials

The following supporting information can be downloaded at: https://www.mdpi.com/article/10.3390/insects14070600/s1, Table S1 [45,46]: List of specimens used in the phylogenetic analysis, their geographic origin (if provided), GenBank accession numbers, and the sources for the sequences downloaded from GenBank. Table S2: List of specimens with 18S used for extraction and amplification during the present study. Their geographic origin, GenBank accession numbers, University of Opole sample numbers, and names of the persons who provided the specimens for analyses are provided. Table S3: Primers used for PCR amplification and sequencing of the nuclear *18S rDNA* gene. File S1: The alignment file of the *18S rDNA* dataset used for the phylogenetic analysis. File S2: The predicted secondary structure models of the *18S rRNA* gene for analysed consensus species. Figure S1: Subdivision of the length-variable regions L (LVRs L) resulting from the alignment of their DNA sequences in five consensus species.
