# Supplementary material for: Tribe Acalyptaini (Hemiptera: Tingidae: Tinginae) Revisited: Can Apomorphies in Secondary and Tertiary Structures of 18S rRNA Length-Variable Regions (LVRs) Support Tribe Validity?
Source: Insects. 2023 Jul 3;14(7):600. doi: 10.3390/insects14070600 (PMC10380217; doi:10.3390/insects14070600)
Supplement: Supplementary file 1 [file insects-14-00600-s001.zip › File S2.pdf]

File S2

The predicted secondary structure models of the 18S rRNA gene for analysed consensus species; for the explanation, see Figure 1. LVRs are indicated in red.

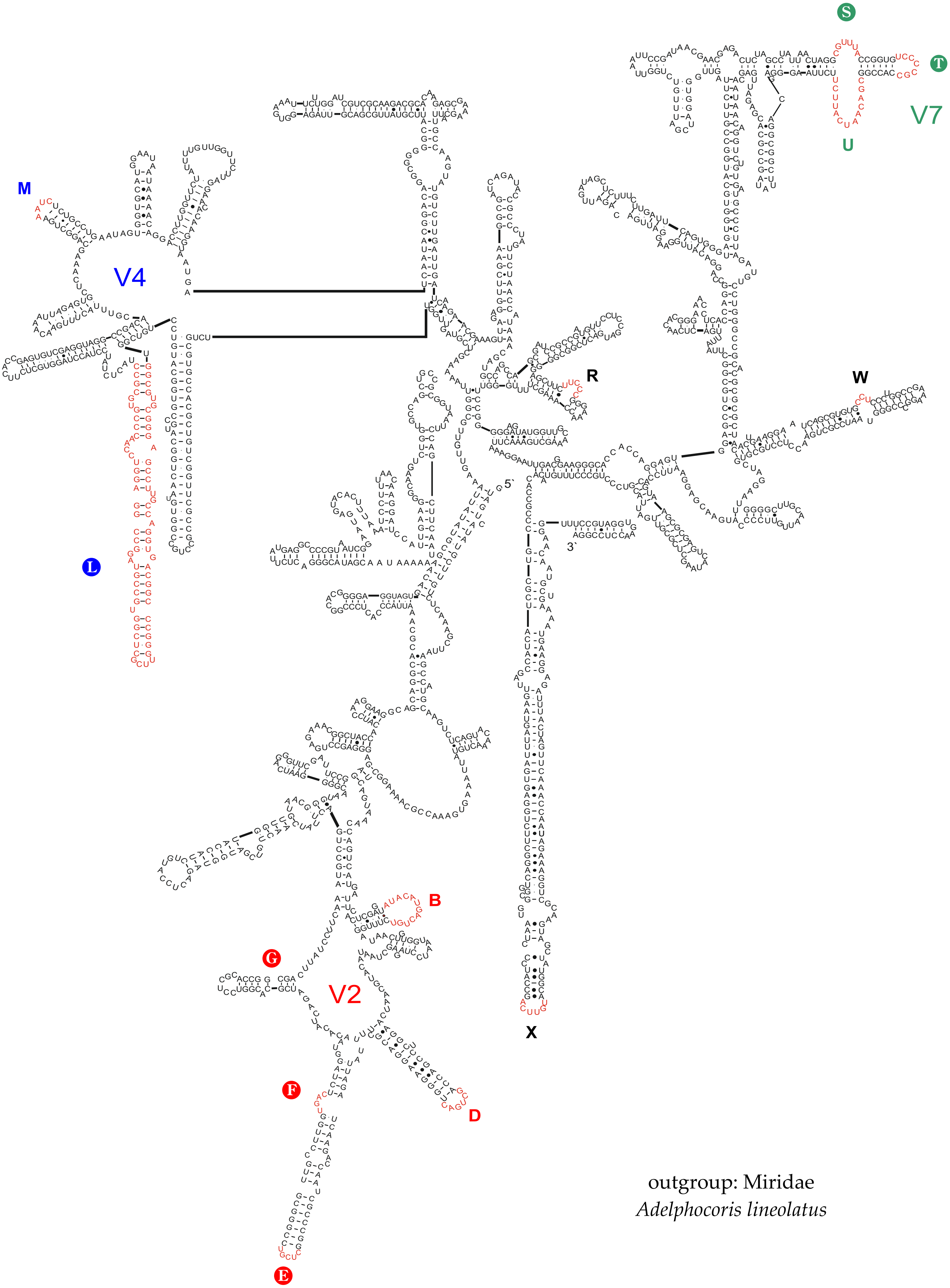

outgroup: Miridae
Adelphocoris lineolatus

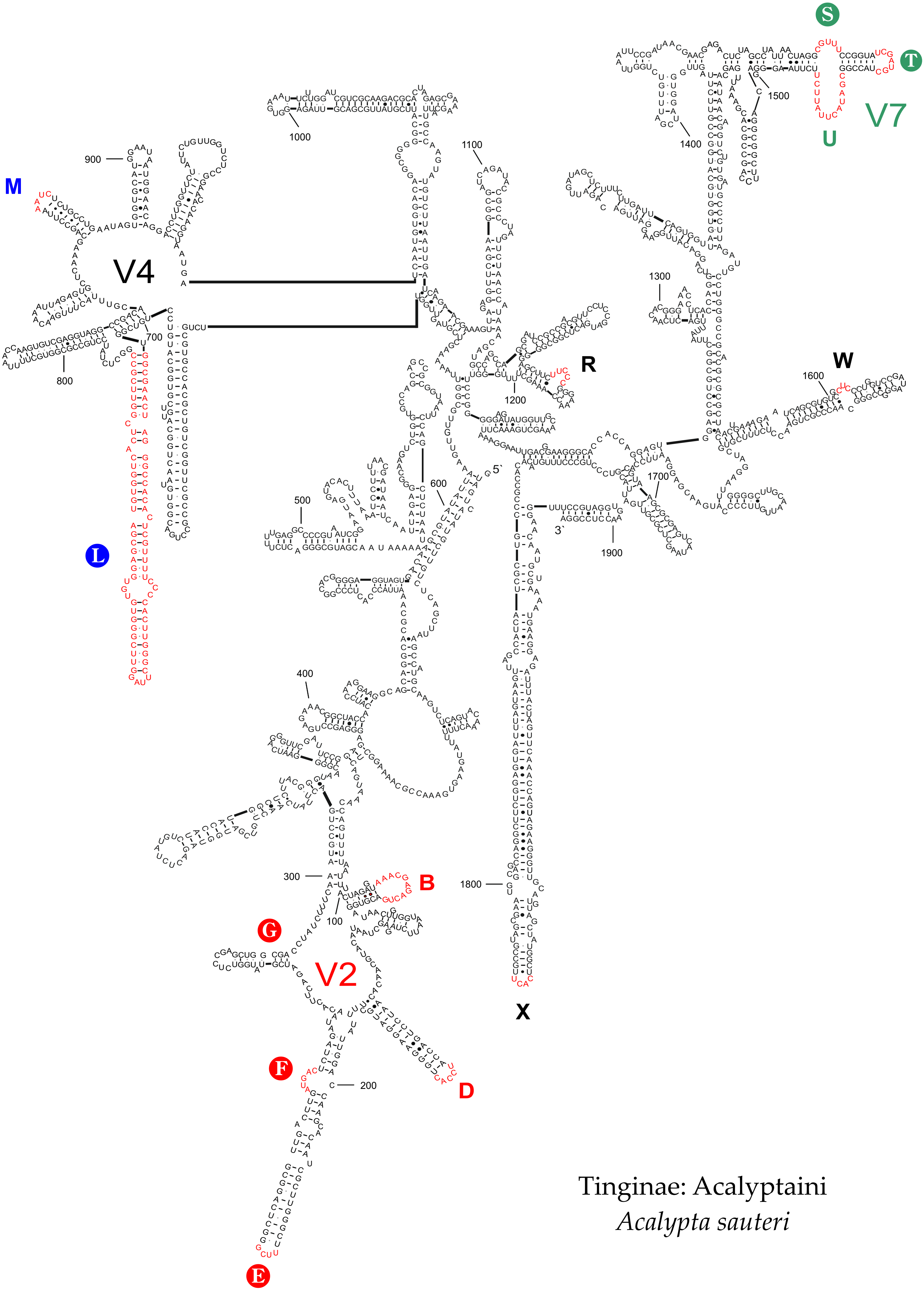

Tinginae: Acalyptaini
Acalypta sauteri

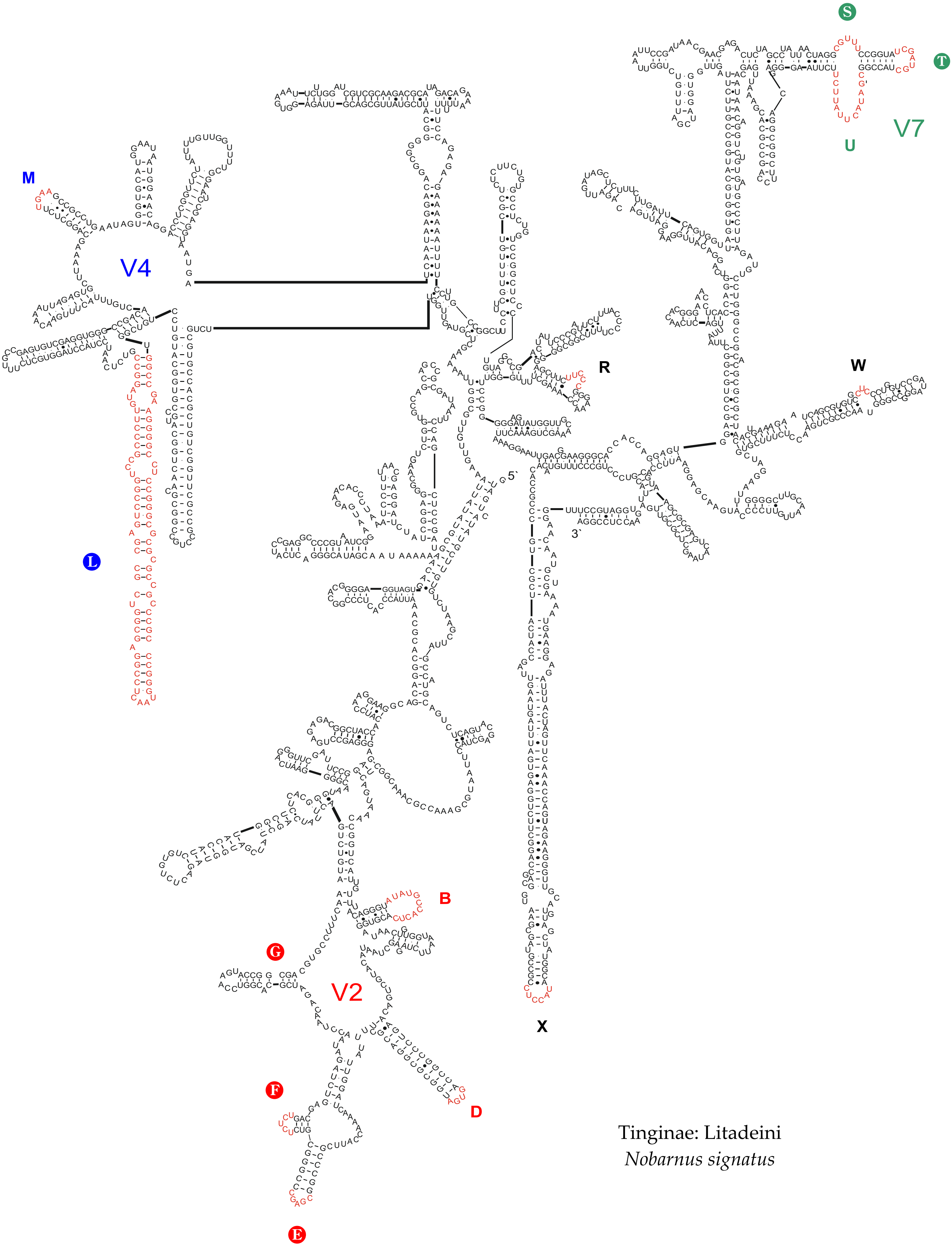

Tinginae: Litadeini
Nobarnus signatus

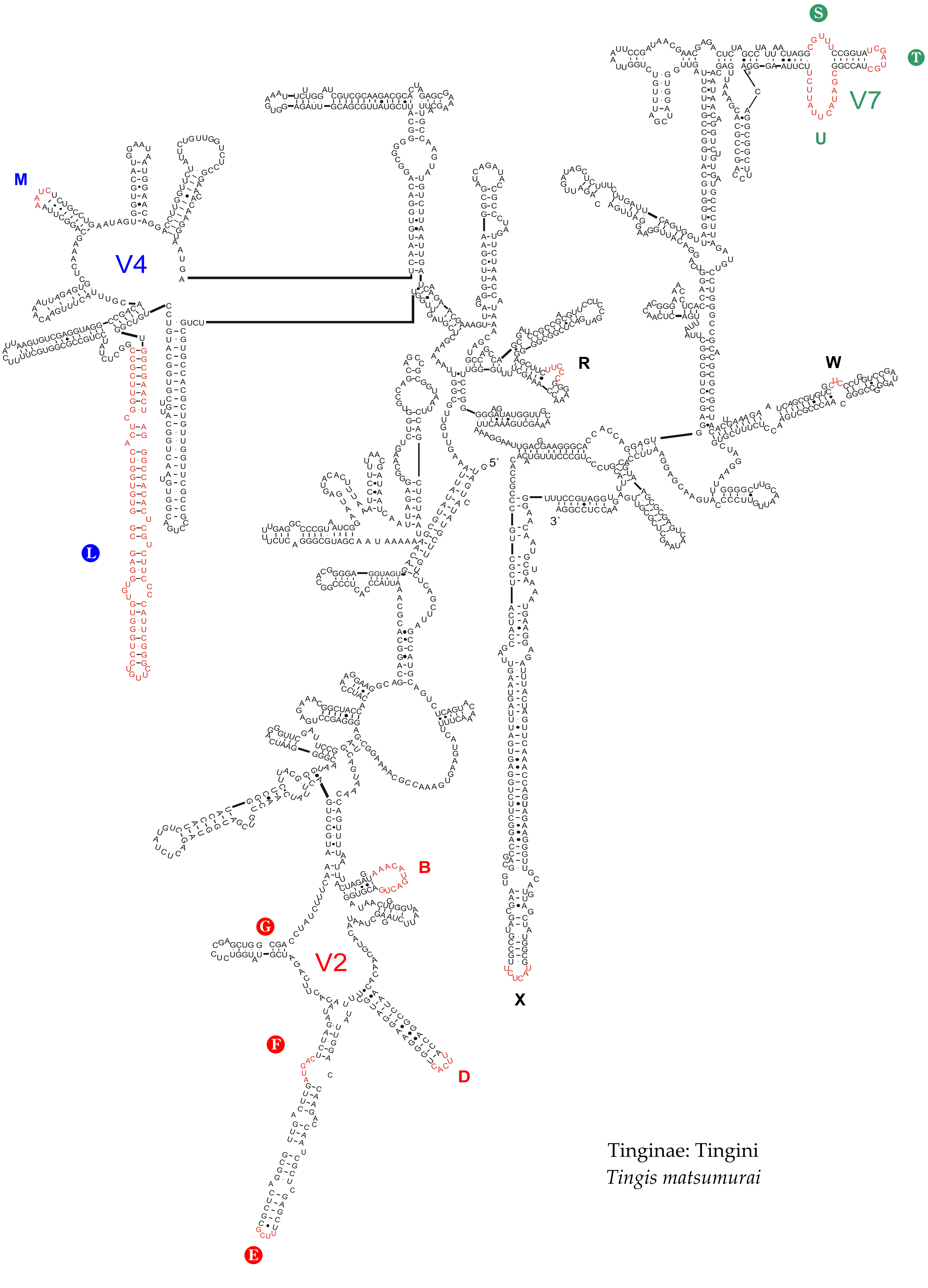

Tinginae: Tingini
Tingis matsumurai

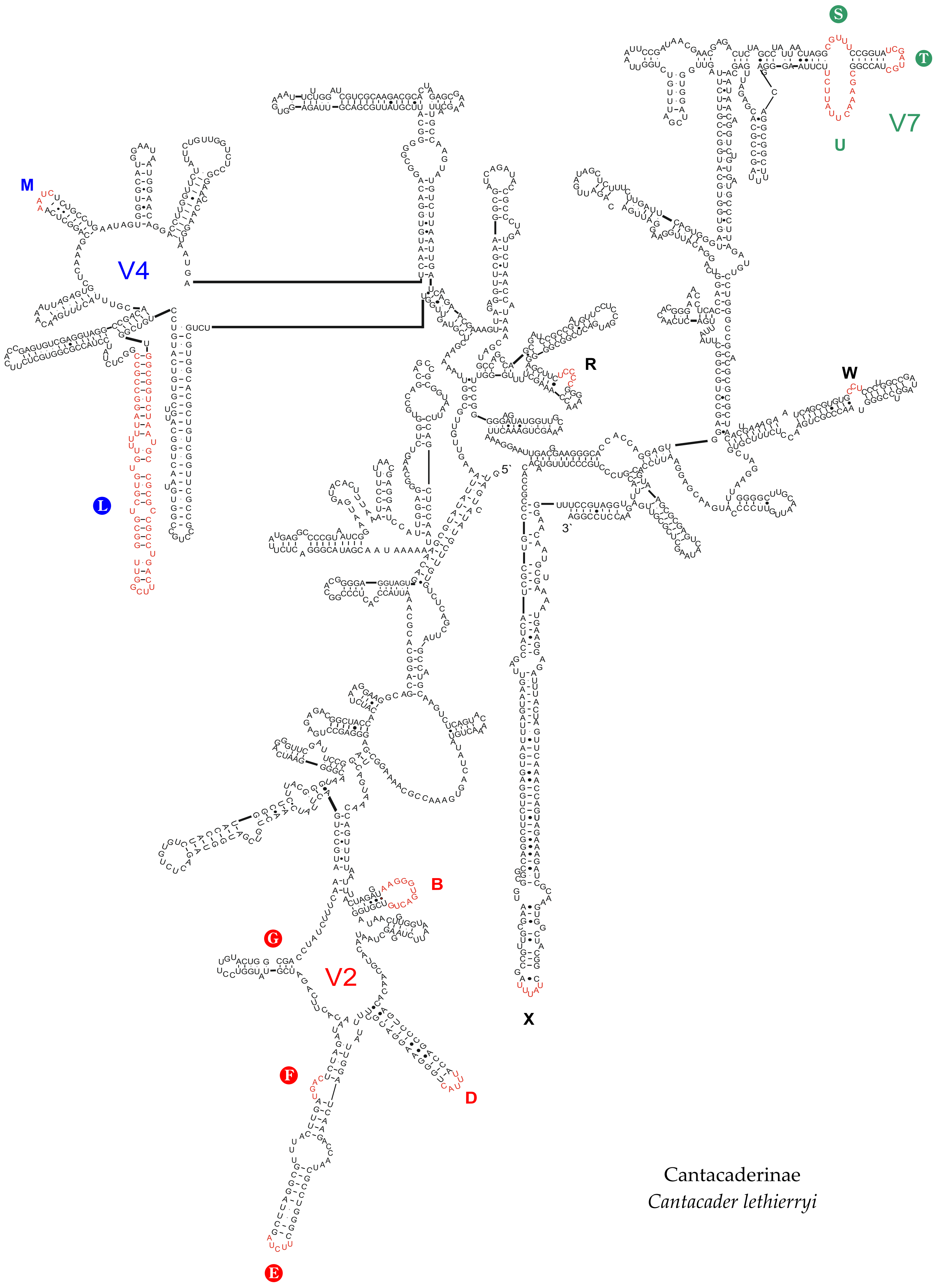

Cantacaderinae
Cantacader lethierryi
