# Supplementary material for: Tribe Acalyptaini (Hemiptera: Tingidae: Tinginae) Revisited: Can Apomorphies in Secondary and Tertiary Structures of 18S rRNA Length-Variable Regions (LVRs) Support Tribe Validity?
Source: Insects. 2023 Jul 3;14(7):600. doi: 10.3390/insects14070600 (PMC10380217; doi:10.3390/insects14070600)
Supplement: Supplementary file 1 [file insects-14-00600-s001.zip › Table S1.pdf]

Table S1. List of specimens used in the phylogenetic analysis, their geographic origin (if provided), GenBank accession numbers, and the sources for the sequences downloaded from GenBank.

Infraorder	Superfamily	Family	Species/subspecies	Geographic origin (year collected, if originally provided)	GenBank accesion numbers for 18S rDNA	Source
**Ingroup**						
Cimicomorpha	Miroidea	Tingidae: Tinginae	*Acalypta miyamotoi* Takeya, 1962	Japan (2011)	OR022068	present study
			Acalypta sauteri Drake, 1942	Japan (2011)	OR022069	present study
			Copium clavicorne (Linnaeus, 1758)	Ukraine (2010)	OR022071	present study
			Corythucha ciliata (Say, 1832)	China	KJ461201	[45]
			Derephysia foliacea (Fallén, 1807)	Poland (2010)	OR022072	present study
			Dictyonota strichnocera Fieber, 1844	Slovakia (2011)	OR022074	present study
			Dictyla humuli (Fabiricius, 1794)	Poland (2010)	OR022073	present study
			Kalama tricornis (Schrank, 1801)	Poland (2008)	OR022075	present study
			Lasiacantha capucina capucina (Germar, 1837)	Poland (2010)	OR022076	present study
			Metasalis populi (Takeya, 1932)	China	KJ461270	[45]
			Nobarnus signatus (Distant, 1920)	New Caledonia (2008)	OR022077	present study
			Oncochila scapularis (Fieber, 1844)	Poland (2010)	OR022078	present study
			Physatocheila fieberi (Scott, 1874)	Japan (2011)	OR022079	present study
			Pseudacysta perseae (Heidemann, 1908)	French Guiana	KM278220	[direct submission to GenBank, unpublished]
			Stephanitis nashi nashi Esaki & Takeya, 1931	Japan (2011)	OR022081	present study
			Stephanitis takeyai Drake & Maa, 1955	Japan (2011)	OR022082	present study
			Tingis matsumurai Takeya, 1962	Japan (2011)	OR022083	present study
		Tingidae: Cantacaderinae	*Cantacader lethierryi* Scott, 1874	Japan (2011)	OR022070	present study
			Pseudophatnoma laosana B. Lis, 1999	Thailand (2013)	OR022080	present study
**Outgroup**						
Cimicomorpha	Miroidea	Miridae	*Adelphocoris lineolatus* (Goeze, 1778)	China	KJ461200	[45]
			Lygus hesperus Knight, 1917	USA (1993)	U06476	[46]
	Naboidea	Nabidae	*Nabis sinicus* (Hsiao, 1964)	China	KJ461288	[45]
