# Supplementary material for: Tribe Acalyptaini (Hemiptera: Tingidae: Tinginae) Revisited: Can Apomorphies in Secondary and Tertiary Structures of 18S rRNA Length-Variable Regions (LVRs) Support Tribe Validity?
Source: Insects. 2023 Jul 3;14(7):600. doi: 10.3390/insects14070600 (PMC10380217; doi:10.3390/insects14070600)
Supplement: Supplementary file 1 [file insects-14-00600-s001.zip › Table S2.pdf]

Table S2. List of specimens with 18S rDNA used for extraction and amplification during the present study. Their geographic origin, GenBank accession numbers, University of Opole sample numbers, and names of the persons who provided the specimens for analyses are provided. All newly sequenced specimens were identified to species by the first author (BAL). Acronyms for the persons who collected or/and provided the specimens for analyses are, as follows: AW (Andrzej Wolski, Institute of Biology, University of Opole, Opole, Poland), BL (Barbara Lis, Institute of Biology, University of Opole, Opole, Poland), MM (Miłosz Mazur, Institute of Biology, University of Opole, Opole, Poland), SK (Shin-ichi Kudo, Department of Biology, Naruto University of Education, Naruto,Tokushima, Japan).

**Family**	**Subfamily**	**Tribe**	**Species/subspecies**	**Geographic origin (year collected, if originally provided)**	**GenBank accesion numbers for 18S rDNA**	**University of Opole sample numbers for newly sequenced species (person who provided the specimen)**
Tingidae	Tinginae	Acalyptaini	*Acalypta miyamotoi* Takeya, 1962	Japan (2011)	OR022068	T47 (SK)
			Acalypta sauteri Drake, 1942	Japan (2011)	OR022069	T48 (SK)
			Derephysia foliacea (Fallén, 1807)	Poland (2010)	OR022072	T48 (BL)
			Dictyonota strichnocera Fieber, 1844	Slovakia (2011)	OR022074	DS2 (MM)
			Kalama tricornis (Schrank, 1801)	Poland (2008)	OR022075	T3 (BL)
			Recaredus rex Distant, 1909	Ghana (1965)	Extraction and amplification failed.	RR63
		Litadeini	*Nobarnus signatus* (Distant, 1920)	New Caledonia (2008)	OR022077	T10
		Tingini	*Copium clavicorne* (Linnaeus, 1758)	Ukraine (2010)	OR022071	T33 (MM)
			Dictyla humuli (Fabiricius, 1794)	Poland (2010)	OR022073	T42 (BL)
			Lasiacantha capucina capucina (Germar, 1837)	Poland (2010)	OR022076	T38 (BL)
			Oncochila scapularis (Fieber, 1844)	Poland (2010)	OR022078	T39 (BL)
			Physatocheila fieberi (Scott, 1874)	Japan (2011)	OR022079	T54 (SK)
			Stephanitis nashi nashi Esaki & Takeya, 1931	Japan (2011)	OR022081	T49 (SK)
			Stephanitis takeyai Drake & Maa, 1955	Japan (2011)	OR022082	T51 (SK)
			Tingis matsumurai Takeya, 1962	Japan (2011)	OR022083	T52 (SK)
	Cantacaderinae	Cantacaderini	*Cantacader lethierryi* Scott, 1874	Japan (2011)	OR022070	T46 (SK)
			Pseudophatnoma laosana B. Lis, 1999	Thailand (2013)	OR022080	T59 (AW)
