# Supplementary material for: Tribe Acalyptaini (Hemiptera: Tingidae: Tinginae) Revisited: Can Apomorphies in Secondary and Tertiary Structures of 18S rRNA Length-Variable Regions (LVRs) Support Tribe Validity?
Source: Insects. 2023 Jul 3;14(7):600. doi: 10.3390/insects14070600 (PMC10380217; doi:10.3390/insects14070600)
Supplement: Supplementary file 1 [file insects-14-00600-s001.zip › Table S3.pdf]

Table S3. Primers used for PCR amplification and sequencing of the nuclear 18S rDNA gene.

Primers		Sequence (5′→3′)	Source
**1st set of primers**	1F	TAC CTG GTT GAT CCT GCC AGT AG	[18]
	5R	CTT GGC AAA TGC TTT CGC	[18]
	3F	GTT CGA TTC CGG AGA GGG A	[18]
	18Sbi	GAG TCT CGT TCG TTA TCG GA	[19]
	5F	GCG AAA GCA TTT GCC AAG AA	[18]
	9R	GAT CCT TCC GCA GGT TCA CCT AC	[18]
**2nd set of primers**	Ns1	GTAGTCATATGCTTGTCTC	[21–23]
	18SP3	GGTTAGAACTAGGGCGGTATCT	[20,22]
	18SP5	CAAGAACGAAAGTTAGAGGT	[23]
	Ns8	TCCGCAGGTTCACCTACGGA	[21–23]
