# Supplementary figures and images for: Tribe Acalyptaini (Hemiptera: Tingidae: Tinginae) Revisited: Can Apomorphies in Secondary and Tertiary Structures of 18S rRNA Length-Variable Regions (LVRs) Support Tribe Validity?
Source: Insects. 2023 Jul 3;14(7):600. doi: 10.3390/insects14070600 (PMC10380217; doi:10.3390/insects14070600)

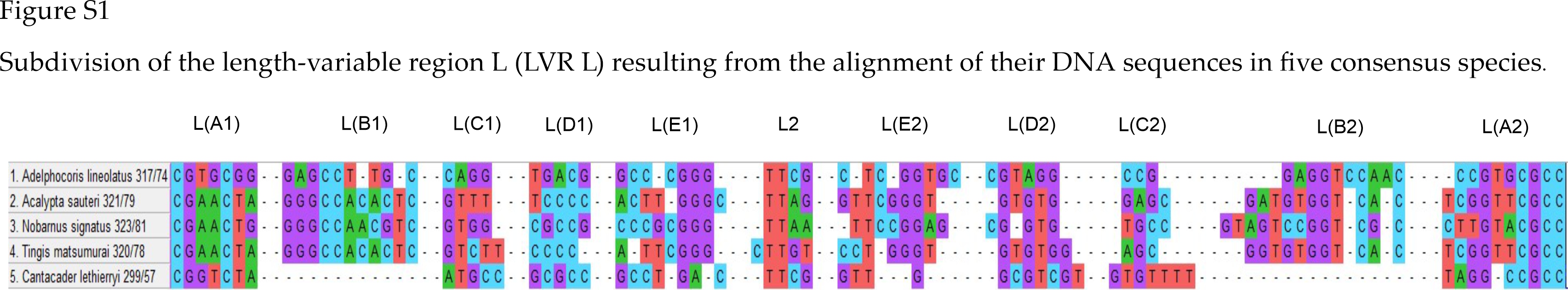

Supplement: Supplementary file 1 [file insects-14-00600-s001.zip › Figure S1.jpg]
